# Injection therapy for carpal tunnel syndrome: A systematic review and network meta-analysis of randomized controlled trials

**DOI:** 10.1371/journal.pone.0303537

**Published:** 2024-05-16

**Authors:** Fu-An Yang, Hsun-Yi Wang, Tien-Yu Kuo, Chih-Wei Peng, Tsan-Hon Liou, Reuben Escorpizo, Hung-Chou Chen

**Affiliations:** 1 Department of Physical Medicine and Rehabilitation, Far Eastern Memorial Hospital, New Taipei City, Taiwan; 2 Department of Internal Medicine, China Medical University Hospital, Taichung, Taiwan; 3 School of Medicine, College of Medicine, Taipei Medical University, Taipei, Taiwan; 4 Department of General Medicine, Kaohsiung Medical University Hospital, Kaohsiung Medical University, Kaohsiung, Taiwan; 5 Department of Physical Medicine and Rehabilitation, Shuang Ho Hospital, Taipei Medical University, New Taipei City, Taiwan; 6 School of Gerontology Health Management, College of Nursing, Taipei Medical University, Taipei, Taiwan; 7 School of Biomedical Engineering, College of Biomedical Engineering, Taipei Medical University, Taipei, Taiwan; 8 Department of Physical Medicine and Rehabilitation, School of Medicine, College of Medicine, Taipei Medical University, Taipei, Taiwan; 9 Department of Rehabilitation and Movement Science, College of Nursing and Health Sciences, University of Vermont, Burlington, VT, United States of America; 10 Swiss Paraplegic Research, Nottwil, Switzerland; 11 Center for Evidence-Based Health Care, Shuang Ho Hospital, Taipei Medical University, Taipei, Taiwan; AIIMS: All India Institute of Medical Sciences, INDIA

## Abstract

Various injectants are available for the treatment of carpal tunnel syndrome. This systematic review and network meta-analysis was conducted to investigate the effectiveness of different injection therapies in alleviating the symptoms of carpal tunnel syndrome. Various databases were searched for relevant studies from inception until May 10, 2023. Eligible studies were identified using the patient (P), intervention (I), comparison (C), and outcomes (O) model, which involved (P) participants with carpal tunnel syndrome, (I) an intervention based on injection therapy, (C) the use of placebo or another injectant as a control treatment, and (O) the measurement of clinical and electrodiagnostic outcomes of interest. A total of 18 studies were included in the analysis. The network meta-analysis revealed that platelet-rich plasma is effective in the treatment of carpal tunnel syndrome in terms of symptom and pain relief and functional improvement in both the short and long term, whereas steroids are effective only in the short term. Additionally, injections of dextrose solution may offer long-term pain relief as well as short- and long-term symptom alleviation and functional improvement. The study findings suggest that platelet-rich plasma should be used as the first-line treatment for carpal tunnel syndrome, with dextrose and steroids serving as alternative treatment options.

## Introduction

Carpal tunnel syndrome, the most prevalent entrapment neuropathy affecting the upper extremities, occurs when the median nerve is compressed as it travels the carpal tunnel [1, 2]. The estimated prevalence of carpal tunnel syndrome in the general population is between 1% and 5% [3, 4]. Work-related activities that require a high degree of force and repetition or the use of hand-operated vibrating tools significantly increase the risk of carpal tunnel syndrome [5]. The underlying pathology involves compression of the median nerve [6], leading to nerve ischemia and subsequent impairment of its function [7]. Common symptoms of carpal tunnel syndrome are numbness, paresthesia, pain, tingling, and weakness across the median nerve distal to the carpal tunnel [8]. Carpal tunnel syndrome can be diagnosed not only through clinical evaluation but through electrodiagnostic studies [9, 10]. Such studies have a sensitivity of 56% to 85% and a specificity of 94% to 99% for carpal tunnel syndrome [10].

Patients who are amenable to minimally invasive treatments can undergo injection therapy for symptom relief [2]. Various injectants—including normal saline, corticosteroids, local anesthetics, 5% dextrose in water, and platelet-rich plasma—are available, but the lack of clear information regarding the effectiveness of these injectants poses a challenge in the treatment selection process [11]. Although studies have indicated that injection therapy with various injectants may alleviate the symptoms of carpal tunnel syndrome [11–15], no study has compared these injectants. This gap in research makes it challenging for clinicians to prioritize injectants for patients with carpal tunnel syndrome. To address this research gap, a systematic review and network meta-analysis of randomized control trials was conducted to compare the effectiveness of injection therapies using different injectants in alleviating the symptoms of carpal tunnel syndrome.

The research questions of interest were as follows:

1. Does injection therapy improve the clinical outcomes of patients with carpal tunnel syndrome?
2. Which injectants result in the most favorable clinical outcomes?

## Methods

This review was performed based on the recommendations outlined in the Cochrane Handbook for Systematic Reviews of Interventions [16], and the protocol adhered to the PRISMA extension statement for network meta-analyses [17]. This systematic review was registered prospectively in the International Prospective Register of Systematic Reviews (PROSPERO) database under number CRD42022341841 on July 4, 2022.

This study analyzed randomized controlled trials, including those using a pilot or crossover design. The patient (P), intervention (I), comparison (C), and outcomes (O) model was used to identify eligible studies; the studies selected for inclusion all involved (P) participants with carpal tunnel syndrome, (I) an intervention based on injection therapy with an injectant of interest, (C) the use of placebo or another injectant as a control treatment, and (O) the measurement of electrodiagnostic and clinical outcomes of interest (including Boston Carpal Tunnel Syndrome Questionnaire [BCTQ] scores and pain score).

Patients who underwent operations, were treated with orthosis, or had another neurological disorder were excluded from this study. Additionally, non-peer-reviewed articles, study protocols, conference papers, letters to the editor, and crossover studies without washout periods were excluded from the analysis. No language restrictions were applied in the search strategy.

Literature review, data extraction, and crosschecks were conducted independently following the guidelines of the Preferred Reporting Items for Systematic Reviews and Meta-Analyses [18]. Relevant articles were searched in the Medline database (by using PubMed), Cochrane Central Register of Controlled Trials, and Embase database from inception to May 10, 2023. The search strategy incorporated terms related to carpal tunnel syndrome, injection therapy, and their synonyms (the detailed search strategies are outlined in the S1 Appendix). Where available, refinement functions of the databases were used to filter results and identify randomized controlled trials. Additional articles were identified through a manual search of the reference lists of the relevant articles.

Two reviewers independently evaluated the eligibility of all the titles and abstracts, and disagreements were resolved through discussion. If necessary, a third reviewer was involved. Only randomized controlled trials that compared the effects of different injectants or placebo on the outcomes of interest among patients with carpal tunnel syndrome were included. Subsequently, the full texts of the remaining articles were read in detail to further assess their eligibility.

Two authors individually extracted data from each study by using a structured form, and the characteristics of all eligible studies were summarized in a table. The following parameters were extracted: (1) the basic information of the qualifying studies (first author and publication date); (2) the severity of carpal tunnel syndrome; (3) the demographic, clinical, and treatment characteristics of the patients (e.g., number and mean age of patients in the control and treatment groups); (4) the treatment protocols and regimens and follow-up duration; and (5) the outcome measurements (including the mean and standard deviation of the outcome measurements before and after treatment in the experimental and control groups). If crucial data could not be extracted from an article, an email was sent to the corresponding author to request the data.

The outcome measurements in this study were clinical assessment scores and electrodiagnostic parameters. The clinical assessments of interest were the BCTQ Symptom Severity Scale (BCTQ-SSS), the BCTQ Functional Status Scale (BCTQ-FSS), and a pain assessment. The BCTQ is widely used to assess the severity of carpal tunnel syndrome in clinical practice; it comprises two parts, namely the SSS (11 items) and the FSS (8 items). Each item is rated on a scale from 1 to 5. A higher score indicates more severe symptoms or functional disability [19]. The electrodiagnostic parameters of interest were sensory nerve action potential (SNAP), sensory nerve conduction velocity (SNCV), distal motor latency (DML), and compound muscle action potential (CMAP).

The quality of the included studies was assessed using the Physiotherapy Evidence Database (PEDro) scale, which is a widely used quality assessment tool for evaluating the risk of bias in randomized controlled trials [20]. The PEDro scale scores assigned by two assessors were compared, and differences were resolved through discussion with a third researcher. The rating of PEDro scores items 2 to 11 are summed to obtain a combined total PEDro score between 0 and 10. Item 1, pertaining to external validity, was excluded from the total score because it addresses eligibility criteria reporting separately in the database to ensure that readers are informed [20]. Scores of <4, 4 and 5, 6 to 8, and 9 to 10 are considered poor, fair, good, and excellent, respectively [20]. All articles meeting the inclusion criteria were included in this review irrespective of their PEDro score.

The network meta-analysis was performed using MetaInsight Version 3.1.12 (https://crsu.shinyapps.io/MetaInsight/) [21], a free online cloud computing network meta-analysis tool for researchers. It synthesizes results and provides a rationale by using the R package netmeta (version 0.9–8) [22].

Continuous data were extracted by adjusting the measurements for change from baseline. In cases where articles did not report standard deviations, authors were contacted for clarification, or missing data were estimated using correlation coefficients, in accordance with the guidelines outlined in the Cochrane Handbook for Systematic Reviews of Interventions [16]. The transitivity assumption, essential for network meta-analysis, was evaluated by comparing distributions of clinical and methodological variables that could serve as effect modifiers across treatment comparisons [16]. A random-effects model was used in this network meta-analysis. Head-to-head comparisons of the effectiveness of injection therapies when using different injectants for carpal tunnel syndrome was performed by estimating the standard mean differences in the variables of interest with corresponding 95% credible intervals. To further analyze and rank the effectiveness of different types of injection therapy for patients with carpal tunnel syndrome, the surface under the cumulative ranking curve (SUCRA) was used as an indicator. SUCRA values range from 0% to 100%, with higher values (closer to 100%) indicating a greater likelihood of a therapy being ranked at or near the top and lower values (closer to 0%) suggesting a higher likelihood of a therapy being ranked at or near the bottom in terms of efficacy. The inconsistency in the network was examined using estimates of loop-specific heterogeneity and local incoherence and by evaluating differences in effect sizes between standard meta-analyses (direct comparisons) and through indirect comparisons [16]. Standard mean differences were used to assess the strength of the relationships among variables in a population; standard mean differences of <0.2, 0.2 to 0.5, 0.5 to 0.8, and >0.8 indicated trivial effects with no clinical significance, weak effects, moderate effects, and strong effects, respectively [23]. Follow-ups lasting <3 and ≥3 months were defined as short-term and long-term follow-ups, respectively.

Anonymized data not published within this article will be made available by request from any qualified investigator.

## Results

**Fig 1:**
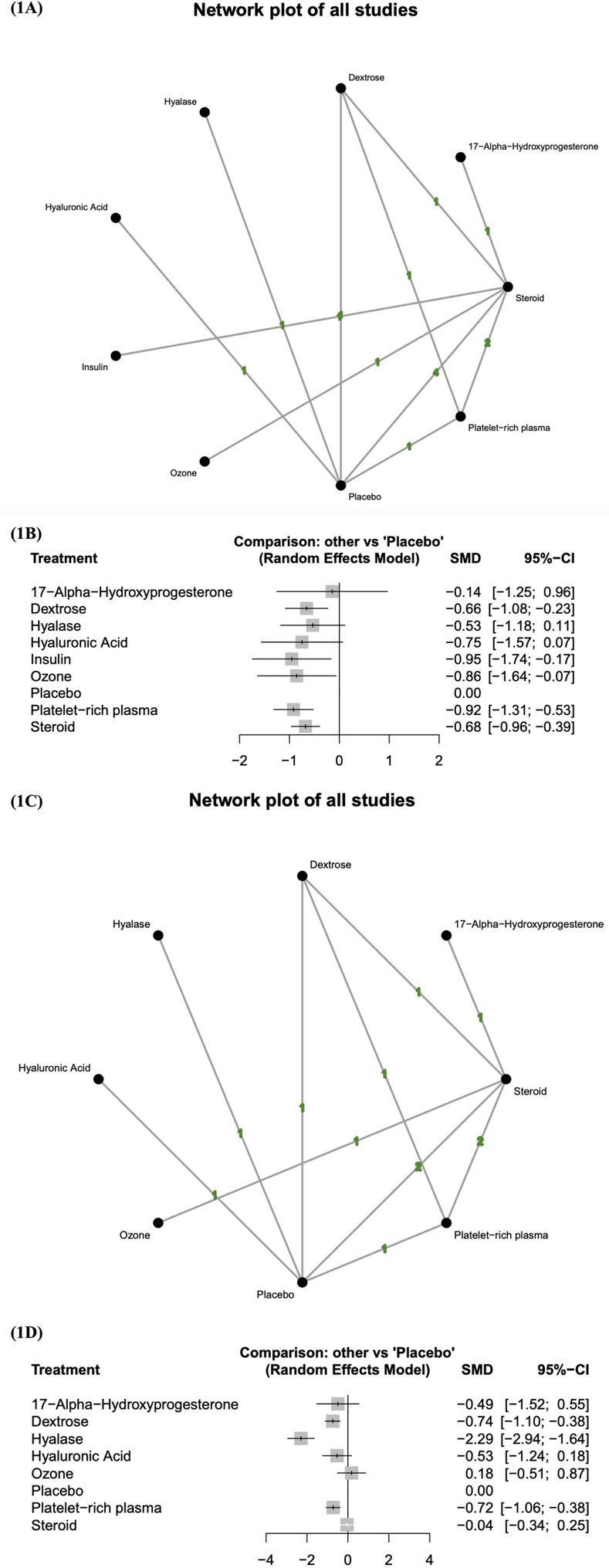
Changes in BCTQ-SSS score. (1A) Network diagram (short-term) displaying at least one placebo-controlled trial for each injectant, except for ozone, insulin, and 17-alpha-hydroxyprogesterone. (1B) Forest plot (short-term) indicating that dextrose, insulin, ozone, platelet-rich plasma, and steroids yielded significantly more favorable outcomes than placebo. (1C) Network diagram (long-term) displaying at least one placebo-controlled trial for each injectant, except for ozone and 17-alpha-hydroxyprogesterone. (1D) Forest plot (long-term) indicating that dextrose, hyalase, and platelet-rich plasma yielded significantly more favorable outcomes than placebo. SMD, standard mean difference; CI, credible interval; BCTQ-SSS, Boston Carpal Tunnel Syndrome Questionnaire Symptom Severity Scale.

The use of the search terms listed in the S1 Appendix yielded an initial set of 2699 studies. Of these, 719 duplicates were excluded using EndNote X9 [24]. Subsequently, 1686 studies that did not meet the inclusion criteria, as observed upon screening their titles and abstracts, were also excluded. Upon further review, 10 additional studies lacking full texts were excluded, leaving 284 studies for full-text screening. Of these, 183 did not meet the inclusion criteria, 3 were review articles, 3 did not report randomized controlled trials, 59 were study protocols, 8 involved studies evaluating combinations of injection therapy and other treatments, 5 were not peer-reviewed, 4 lacked sufficient data, and 1 reported a study involving patients with underlying disease. Finally, 18 articles, with a total of 991 participants, were included in this meta-analysis [25–42]. A PRISMA flowchart illustrating the selection process and numbers of articles, with reasons for study exclusion in each step of the meta-analysis [18], is presented in Fig 1 in S2 File.

In the 18 selected randomized controlled trials, various injection treatment protocols were used. Three used dextrose [31, 34, 36], one used hyalase [30], one used insulin [33], 13 used steroid [26, 27, 29, 32–35, 37–39, 41, 42], one used hyaluronic acid [25], one used 17-Alpha-Hydroxyprogesterone [39], four used platelet-rich plasma [28, 29, 31, 32], and one used ozone [27]. Normal saline injection was considered as placebo. Most studies enrolled patients with mild-to-moderate carpal tunnel syndrome [25–27, 29–37, 39, 41, 42], one article included moderate-to-severe carpal tunnel syndrome [28] and two articles did not mention the severity of carpal tunnel syndrome [38, 40]. Table 1 in S1 File summarized the main characteristics of the 18 randomized controlled trials.

Two reviewers independently assessed the quality of the included randomized controlled trials by using the PEDro scale. All the PEDro scores of the included studies were between 5 and 10. On the basis of these scores, 1 trial was categorized as fair [39], 7 were categorized as good [25, 28–33], and 10 were categorized as excellent [26, 27, 34–38, 40–42]. The detailed results for the bias risk assessment are presented in Table 2 in S1 File.

### Short-term changes in BCTQ-SSS score

The network diagram of the studies that measured the short-term effects of injection therapies on BCTQ-SSS score is presented in Fig 1A. At least one placebo-controlled trial was identified for each injectant except for ozone, insulin, and 17-alpha-hydroxyprogesterone. The pooled standard mean differences of the short-term changes in BCTQ-SSS score revealed that dextrose, insulin, ozone, platelet-rich plasma, and steroids resulted in significantly better outcomes than did placebo (Fig 1B). Table 3 in S1 File reveals the results of the pairwise meta-analysis and network meta-analysis on the short-term changes in BCTQ-SSS score. According to the probability rankings, platelet-rich plasma is the most effective injectant, followed by insulin, ozone, hyaluronic acid, steroids, dextrose, hyalase, 17-alpha-hydroxyprogesterone, and placebo.

The network diagram (Fig 1A) contains four triangle loops (dextrose–placebo–steroids, dextrose–placebo–platelet-rich plasma, dextrose–platelet-rich plasma–steroids, and steroids–platelet-rich plasma–placebo), and the loop-specific heterogeneity estimates demonstrated no significant inconsistency between the results of the direct and indirect comparisons except in the comparison of steroids with placebo (Table 4 in S1 File).

### Long-term changes in BCTQ-SSS score

The network diagram of the studies that measured the long-term effects of injection therapies on BCTQ-SSS score is presented in Fig 1C. At least one placebo-controlled trial was identified for each injectant except for ozone and 17-alpha-hydroxyprogesterone. The pooled standard mean differences of long-term changes in BCTQ-SSS score revealed that dextrose, hyalase, and platelet-rich plasma resulted in significantly more favorable clinical outcomes than did placebo (Fig 1D). Table 5 in S1 File presents the results of the pairwise meta-analysis and network meta-analysis on the long-term changes in BCTQ-SSS score. According to the probability rankings, hyalase is the most effective injectant, followed by dextrose, platelet-rich plasma, hyaluronic acid, 17-alpha-hydroxyprogesterone, steroids, placebo, and ozone.

The network diagram (Fig 1C) depicts four triangle loops (dextrose–placebo–steroids, dextrose–placebo–platelet-rich plasma, dextrose–platelet-rich plasma–steroids, and steroids–platelet-rich plasma–placebo), and no significant inconsistency was revealed between the results of the direct and indirect comparisons in the loop-specific heterogeneity estimates (Table 6 in S1 File).

### Short-term changes in BCTQ-FSS score

**Fig 2:**
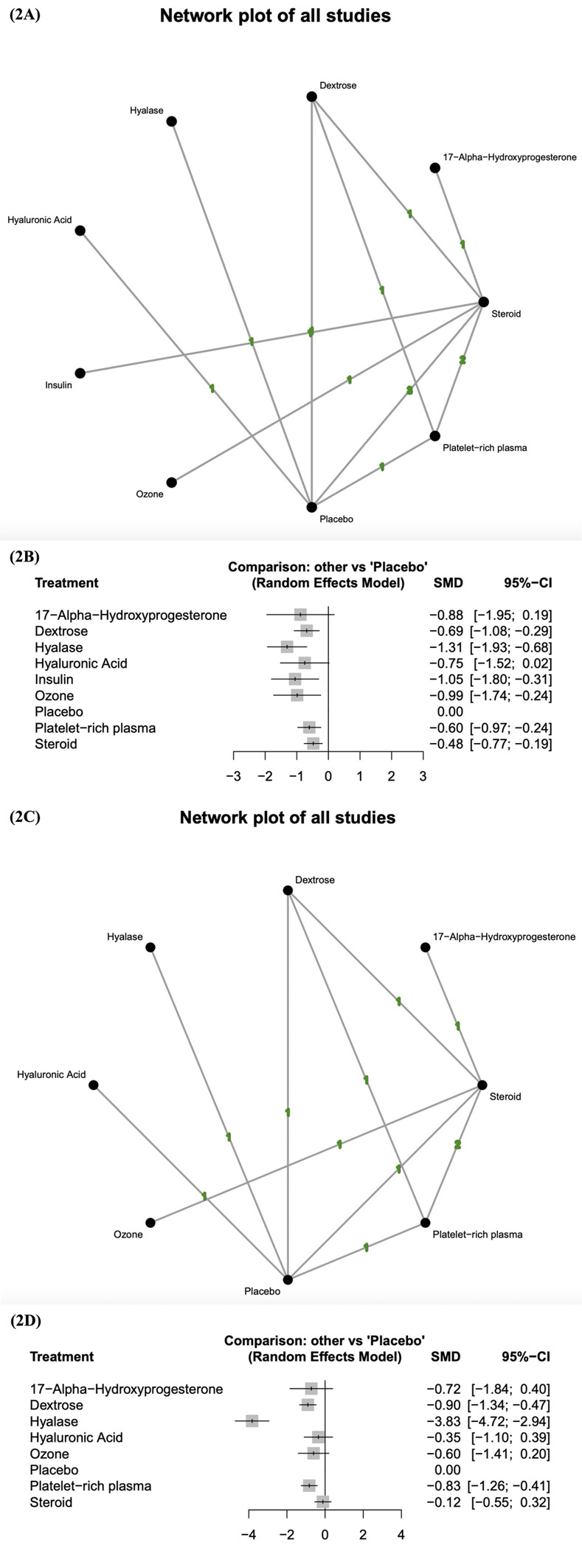
Changes in BCTQ-FSS score. (2A) Network diagram (short-term) displaying at least one placebo-controlled trial for each injectant, except for ozone, insulin, and 17-alpha-hydroxyprogesterone. (2B) Forest plot (short-term) indicating that dextrose, hyalase, insulin, ozone, platelet-rich plasma, and steroids yielded significantly more favorable outcomes than placebo. (2C) Network diagram (long-term) displaying at least one placebo-controlled trial for each injectant, except for ozone and 17-alpha-hydroxyprogesterone. (2D) Forest plot (long-term) indicating that dextrose, hyalase, and platelet-rich plasma yielded significantly more favorable outcomes than placebo. SMD, standard mean difference; CI, credible interval; BCTQ-FSS, Boston Carpal Tunnel Syndrome Questionnaire Functional Status Scale.

The network diagram of the studies that measured the short-term effects of injection therapies on BCTQ-FSS score is presented in Fig 2A. At least one placebo-controlled trial was identified for each injectant except for ozone, insulin, and 17-alpha-hydroxyprogesterone. The pooled standard mean differences of short-term changes in BCTQ-FSS score revealed that dextrose, hyalase, insulin, ozone, platelet-rich plasma, and steroids resulted in significantly better outcomes than did placebo (Fig 2B). Table 7 in S1 File presents the results of the pairwise meta-analysis and network meta-analysis on the short-term changes in BCTQ-FSS score. According to the probability rankings, hyalase is the most effective injectant, followed by insulin, ozone, 17-alpha-hydroxyprogesterone, hyaluronic acid, dextrose, platelet-rich plasma, steroids, and placebo.

The network diagram (Fig 2A) depicts four triangle loops (dextrose–placebo–steroids, dextrose–placebo–platelet-rich plasma, dextrose–platelet-rich plasma–steroids, and steroids–platelet-rich plasma–placebo), and no significant inconsistency was revealed between the results of the direct and indirect comparisons in the loop-specific heterogeneity estimates (Table 8 in S1 File).

### Long-term changes in BCTQ-FSS score

The network diagram of the studies that measured the long-term effects of injection therapies on BCTQ-FSS score is presented in Fig 2C. At least one placebo-controlled trial was identified for each injectant except for ozone and 17-alpha-hydroxyprogesterone. The pooled standard mean differences of long-term changes in BCTQ-FSS score revealed that dextrose, hyalase, and platelet-rich plasma resulted in significantly better outcomes than did placebo (Fig 2D). Table 9 in S1 File presents the results of the pairwise meta-analysis and network meta-analysis on the long-term changes in BCTQ-FSS score. According to the probability rankings, hyalase is the most effective injectant, followed by dextrose, platelet-rich plasma, 17-alpha-hydroxyprogesterone, ozone, hyaluronic acid, steroids, and placebo.

The network diagram (Fig 2C) depicts four triangle loops (dextrose–placebo–steroids, dextrose–placebo–platelet-rich plasma, dextrose–platelet-rich plasma–steroids, and steroids–platelet-rich plasma–placebo), and no significant inconsistency was revealed between the results of the direct and indirect comparisons in the loop-specific heterogeneity estimates (Table 10 in S1 File).

### Short-term changes in pain score

**Fig 3:**
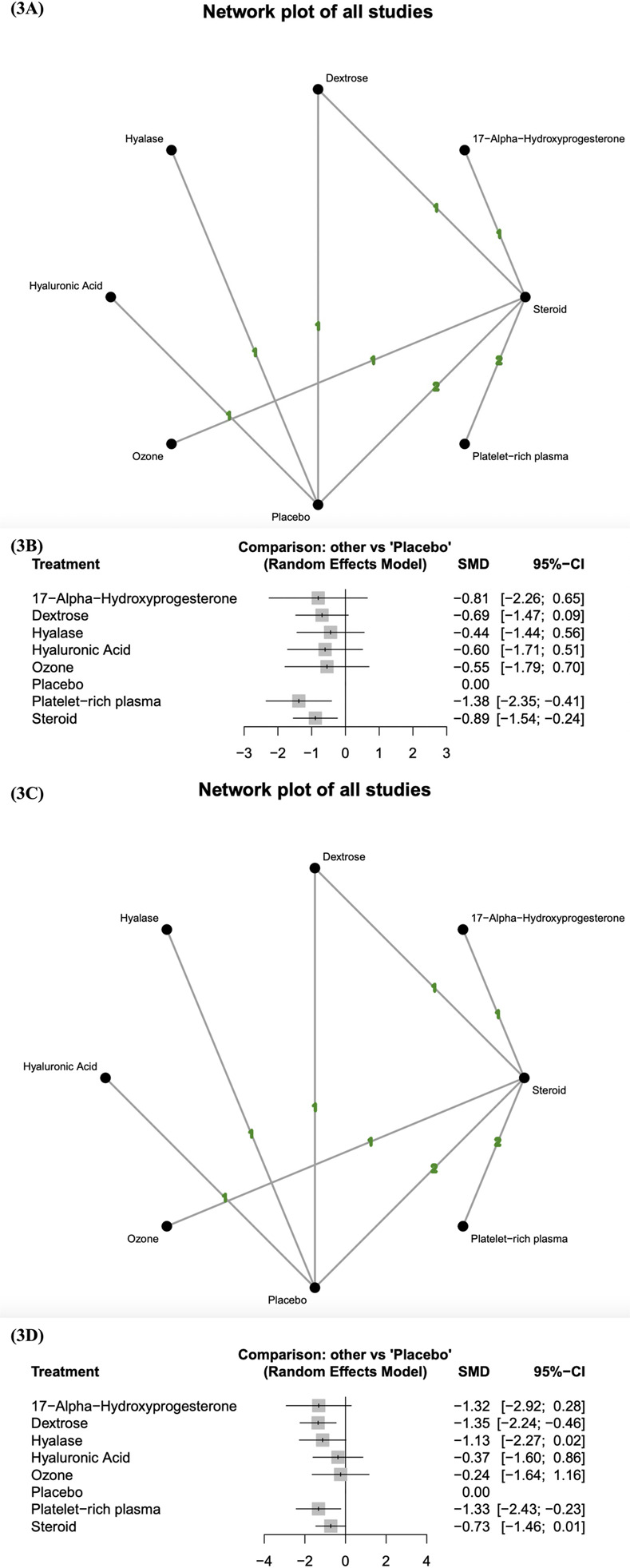
Changes in pain score. (3A) Network diagram (short-term) displaying at least one placebo-controlled trial for each injectant, except for ozone, platelet-rich plasma, and 17-alpha-hydroxyprogesterone. (3B) Forest plot (short-term) indicating that platelet-rich plasma and steroids yielded significantly more favorable outcomes than placebo. (3C) Network diagram (long-term) displaying at least one placebo-controlled trial for each injectant, except for ozone, platelet-rich plasma, and 17-alpha-hydroxyprogesterone. (3D) Forest plot (long-term) indicating that dextrose and platelet-rich plasma yielded significantly more favorable outcomes than placebo. SMD, standard mean difference; CI, credible interval.

The network diagram of the studies that measured the short-term effects of injection therapies on pain score is presented in Fig 3A. At least one placebo-controlled trial was identified for each injectant except for ozone, platelet-rich plasma, and 17-alpha-hydroxyprogesterone. The pooled standard mean differences of short-term changes in pain score revealed that platelet-rich plasma and steroids resulted in significantly better outcomes than did placebo (Fig 3B). Table 11 in S1 File presents the results of the pairwise meta-analysis and network meta-analysis on the short-term changes in pain score. According to the probability rankings, platelet-rich plasma is the most effective injectant, followed by steroids, 17-alpha-hydroxyprogesterone, dextrose, hyaluronic acid, ozone, hyalase, and placebo.

The network diagram (Fig 3A) depicts one triangle loop (dextrose–placebo–steroids), and no significant inconsistency was revealed between the results of the direct and indirect comparisons in the loop-specific heterogeneity estimates (Table 12 in S1 File).

### Long-term changes in pain score

The network diagram of the studies that measured the long-term effects of injection therapies on pain score is presented in Fig 3C. At least one placebo-controlled trial was identified for each injectant except for ozone, platelet-rich plasma, and 17-alpha-hydroxyprogesterone. The pooled standard mean differences of long-term changes in pain score revealed that dextrose and platelet-rich plasma resulted in significantly better outcomes than did placebo (Fig 3D). Table 13 in S1 File presents the results of the pairwise meta-analysis and network meta-analysis on the long-term changes in pain score. According to the probability rankings, dextrose is the most effective injectant, followed by platelet-rich plasma, 17-alpha-hydroxyprogesterone, hyalase, steroids, hyaluronic acid, ozone, and placebo.

The network diagram (Fig 3C) depicts one triangle loop (dextrose–placebo–steroids), and no significant inconsistency was revealed between the results of the direct and indirect comparisons in the loop-specific heterogeneity estimates (Table 14 in S1 File).

### Short-term changes in electrodiagnostic parameters

The patients’ electrodiagnostic parameters—namely SNCV, SNAP, DML, and CMAP—were analyzed at short-term follow-ups. The corresponding network diagrams and forest plots are presented in Figs 2–5 in S2 File. Steroids yielded significantly more favorable outcomes in SNCV compared with placebo [SMD = 0.34, 95% CI = (0.03, 0.65)]. The other outcomes did not differ significantly among the injectants (Figs 2–5 in S2 File).

### Long-term changes in electrodiagnostic parameters

The patients’ electrodiagnostic parameters at long-term follow-ups were examined. The corresponding network diagrams and forest plots are presented in Figs 6–9 in S2 File. Compared with placebo, dextrose [SMD = 0.37, 95% CI = (0.02, 0.73)], hyalase [SMD = 0.63, 95% CI = (0.11, 1.15)], platelet-rich plasma [SMD = 0.46, 95% CI = (0.11, 0.82)], and steroids [SMD = 0.32, 95% CI = (0.02, 0.63)] yielded significantly more favorable outcomes in SNCV, whereas hyalase [SMD = −1.03, 95% CI = (−1.57, −0.49)] yielded significantly more favorable outcomes in DML. The other outcomes did not differ significantly among the injectants (Figs 6–9 in S2 File).

### Adverse effects

Of the included articles, 15 reported whether side effects occurred during the intervention period [25–27, 31–42]. Most of these studies reported no adverse events during intervention or follow-up [25–27, 31, 33–36, 38–40]. The most common adverse effect was injection site pain after the injection was administered [32, 37, 42]. Only one article reported a steroid flare (a delayed transient increase in pain after an injection; such flares have led to crystal-induced synovitis) that occurred after the administration of a corticosteroid [41].

## Discussion

Various injectants have been used to treat carpal tunnel syndrome. In the present study, the effectiveness of these injectants was investigated through a network meta-analysis of randomized control trials. Platelet-rich plasma was identified to be the most effective injectant for short-term BCTQ-SSS improvement and short-term pain relief. Hyalase exhibited superiority in long-term BCTQ-SSS, short-term BCTQ-FSS, and long-term BCTQ-FSS improvement. Furthermore, dextrose demonstrated the highest efficacy in long-term pain alleviation. The clinical implications of the findings are as follows. First, platelet-rich plasma is effective in alleviating symptoms and pain associated with carpal tunnel syndrome and improving function over both the short and long term. Second, dextrose is effective in terms of symptom and pain relief and functional improvement in both the short and long term, except for pain relief in the short term. Third, steroids are effective in terms of symptom and pain relief and functional improvement in the short term, but their long-term effects are not significant. These findings all correspond to moderate to strong clinical effects. Fourth, in the short term, steroids yielded significantly more favorable outcomes in SNCV, whereas in the long term, dextrose, hyalase, platelet-rich plasma, and steroids yielded significantly more favorable outcomes in SNCV, with hyalase demonstrating superiority in DML. In this network meta-analysis, although platelet-rich plasma was not consistently identified as the most effective injectant for all outcomes of interest, it consistently yielded the most significant improvements across all clinical outcomes over both the short and long term. As a result, platelet-rich plasma should be used as first-line treatment for carpal tunnel syndrome, and dextrose and steroids may serve as alternative treatments.

Platelet-rich plasma is the processed liquid fraction of autologous peripheral blood with a platelet concentration [43]. The underlying scientific rationale for platelet-rich plasma therapy is that an injection of concentrated platelets at sites of injury may stimulate tissue repair through the release of numerous biologically active factors and adhesion proteins that induce initiation of the hemostatic cascade, synthesis of new connective tissue, and revascularization [44]. A recent meta-analysis demonstrated that platelet-rich plasma is effective in alleviating the symptoms of carpel tunnel syndrome but lacks long-term efficacy [45]. In this network meta-analysis, platelet-rich plasma consistently demonstrated the most favorable outcomes across all clinical parameters, over both the short and long term. Therefore, platelet-rich plasma is recommended as the first-line treatment for carpal tunnel syndrome.

Prolotherapy involves the injection of an irritant (typically a dextrose solution) and appears to be a promising treatment for managing chronic painful musculoskeletal conditions [46]. There is incomplete understood mechanism of action of prolotherapy, but the most widely accepted theory is that prolotherapy initiates a local inflammatory cascade, leads to tissue proliferation and remodeling, is thought to be involved in the healing process [47]. In carpal tunnel syndrome, characterized by nerve compression and traction, leading to intraneural microcirculation disorders and alterations in the connective tissue support [48], prolotherapy may expedite the regeneration process and promote tissue repair posttreatment [49]. The analysis suggests that injections of dextrose solution could be effective in providing long-term pain relief as well as short- and long-term alleviation of symptoms and improvement of function.

Local corticosteroid injections have been widely used in clinical practice as a nonoperative treatment for carpal tunnel syndrome because of their anti-inflammatory effects [50]. Corticosteroid injections are effective for short-term (1 to 3 months) relief of the symptoms of carpal tunnel syndrome; however, their long-term benefits are less certain [37, 51]. Previous studies have suggested that steroids typically offer relief for approximately 1–2 weeks after they are absorbed from the joint, metabolized by the liver, and excreted by the kidney [52]. Unlike prolotherapy, corticosteroids offer only anti-inflammatory effects without promoting tissue regeneration. Therefore, the findings are compatible with previous research. The results of the analysis indicate that corticosteroid injections are effective in terms of symptom and pain relief as well as functional improvement in the short term but not in the long term.

Hyaluronic acid is a glycosaminoglycan that occurs naturally within the synovial fluid of the joints, lubricating the joints and protecting the cartilage from mechanical degradation [53]. Its viscoelastic properties, as evidenced in various studies, prevent adhesions and nerve scar formation, thus facilitating nerve repair and regeneration [25, 54, 55]. Conversely, hyalase, an enzyme catalyzing the degradation of hyaluronic acid, promotes remyelination in demyelinating lesions [30]. 17-Alpha-hydroxyprogesterone, an endogenous progestogen related to progesterone, has neuroprotective effects [39]. The peripheral nerves have numerous receptors for nerve growth factor (a member of the insulin-like growth factor 1 family) and insulin [56], both of which promote neuronal growth and regeneration and may be key to the ability of local insulin injections to restore nerve function [57]. Ozone is a re-emerging substance that has bactericidal, immune-modulatory, analgesic, anti-inflammatory, and antioxidative properties and can enhance blood circulation [58]. Although hyaluronic acid, hyalase, 17-alpha-hydroxyprogesterone, insulin, and ozone theoretically hold promise in alleviating the symptoms of carpal tunnel syndrome, their actual efficacy could not be fully assessed in this analysis due to the limited number of included studies. Therefore, future studies should further evaluate the effectiveness of these agents in the treatment of carpal tunnel syndrome.

Although determining that the injectants evaluated in this study were effective in terms of symptom and pain relief as well as functional improvement, limited improvement was observed in terms of electrodiagnostic parameters. The lack of association between clinical outcomes and electrodiagnostic parameters in the present study may have been due to the routine electrodiagnostic testing mainly evaluating large fibers rather than the small sensory fibers that are involved in producing many of the symptoms of carpal tunnel syndrome [59]. This suggests that electrodiagnostic testing may have limited ability to predict the therapeutic outcomes for patients with carpel tunnel syndrome following conservative treatment [32].

Recent advancements have been made in injection therapy techniques. Ultrasound guidance offers accurate, real-time imaging of the wrist structure to facilitate direct drug injection into the carpal tunnel [12]. Ultrasound-guided injections yielded better results than did landmark-guided injections in one study [12]. In addition to ultrasound-guided injection, hydrodissection has been receiving attention as a therapy for carpal tunnel syndrome [60]. Hydrodissection is safe and effective and treats carpal tunnel syndrome by producing not only a mechanical effect (releasing and decompressing entrapped nerves) but also a pharmacological effect (relieving pain and promoting recovery through numerous mechanisms) [11]. Among the studies included in the present analysis, 11 involved the use of ultrasound-guided techniques [25, 27, 28, 30–36, 39], and 8 involved the use of hydrodissection [25, 28, 30, 31, 34–36, 40]. However, due to variations in injectants used, the efficacy of these techniques could not be compared. The relative efficacy of these techniques should be further evaluated in future studies.

### Study strengths and limitations

The present study has several strengths. First, this is the first network meta-analysis of randomized controlled trials to focus on the efficacy of various injectants in the treatment of carpal tunnel syndrome. Second, based on the findings, practical recommendations were formulated regarding the optimal use of different injectants in clinical practice. Third, network meta-analyses allow for estimates of the relative effects between any pair of interventions in a network to be determined, often providing more precise results compared with single direct or indirect estimates. Fourth, the study extensively utilized multiple major databases to identify randomized control trials and imposed no language restrictions. Finally, the quality of most of the selected randomized controlled trials (according to their PEDro score) was from good to excellent.

This study also has several limitations. First, considerable variation was observed among the included studies in terms of symptom duration and injectant dosage, which may have influenced the findings related to the effectiveness of the interventions. Second, only 18 studies were included in the analysis, with a limited number of studies focusing on specific injectants such as hyaluronic acid, hyalase, 17-alpha-hydroxyprogesterone, insulin, and ozone. Therefore, drawing definitive conclusions regarding these injectants on the basis of the available data was challenging. Third, comparing the efficacy of ultrasound-guided injections with hydrodissection or other techniques used in the treatment of carpal tunnel syndrome was challenging. Additional high-quality and large-scale randomized controlled trials are required to address these limitations.

## Conclusion

The study is the first network meta-analysis of randomized controlled trials investigating the efficacy of various injectants used in the treatment of carpal tunnel syndrome. The findings suggest that platelet-rich plasma can be used as the first-line treatment for carpal tunnel syndrome, with dextrose and steroids as viable alternatives when necessary. Future studies should further evaluate the relative efficacy of these injectants for the treatment of carpal tunnel syndrome in terms of symptom and pain relief and functional improvement.

## Supporting information

S1 Appendix Keywords used for searching relevant articles in various electronic databases. (DOCX)

S1 File (DOCX)

S2 File (DOCX)

S1 Checklist PRISMA 2020 checklist. (DOCX)
