# Supplementary material for: Injection therapy for carpal tunnel syndrome: A systematic review and network meta-analysis of randomized controlled trials
Source: PLoS One. 2024 May 16;19(5):e0303537. doi: 10.1371/journal.pone.0303537 (PMC11098370; doi:10.1371/journal.pone.0303537)
Supplement: S1 Appendix — (DOCX) [file pone.0303537.s001.docx]

**Appendix**

Keywords used for searching relevant articles in various electronic databases

| Database | Search terms for query |
| --- | --- |
| **PubMed (started from 1782)** | Search for articles published from database inception to May 10, 2023. |
| #1 | Carpal tunnel |
| #2 | Median nerve |
| #3 | Median neuropathy |
| #4 | Median neuropathies |
| #5 | Median neuritis |
| #6 | Carpal tunnel syndrome [MeSH] |
| #7 | #1 OR #2 OR #3 OR #4 OR #5 OR #6 |
| #8 | Entrapment |
| #9 | Compress |
| #10 | Compressive |
| #11 | Compression |
| #12 | Focal |
| #13 | #8 OR #9 OR #10 OR #11 OR #12 |
| #14 | Neuropathy |
| #15 | Neuropathies |
| #16 | Neuritis |
| #17 | #14 OR #15 OR #16 |
| #18 | #13 AND #17 |
| #19 | #7 OR #18 |
| #20 | Inject* |
| #21 | Steroid |
| #22 | Steroids |
| #23 | Corticosteroid |
| #24 | Corticosteroids |
| #25 | Betamethasone |
| #26 | Triamcinolone acetonide |
| #27 | Methylprednisolone |
| #28 | #21 OR #22 OR #23 OR #24 OR #25 OR #26 OR #27 |
| #29 | Dextrose |
| #30 | Glucose |
| #31 | D5W |
| #32 | Prolotherapy |
| #33 | Prolotherapy [MeSH] |
| #34 | #29 OR #30 OR #31 OR #32 OR #33 |
| #35 | Platelet rich |
| #36 | PRP |
| #37 | Autologous |
| #38 | Platelet rich plasma [MeSH] |
| #39 | #35 OR #36 OR #37 OR #38 |
| #40 | Ozone |
| #41 | Ozone [MeSH] |
| #42 | #40 OR #41 |
| #43 | Hyaluronic |
| #44 | Hyaluronic acid [MeSH] |
| #45 | #43 OR #44 |
| #46 | Hyalase |
| #47 | Progesterone |
| #48 | #20 OR #28 OR #34 OR #39 OR #42 OR #45 OR #46 OR #47 |
| #49 | #19 AND #48 |

| Database | Search terms for query |
| --- | --- |
| **Excerpta Medica Database (EMBASE; started from 1947)** | Search for articles published from database inception to May 10, 2023. |
| #1 | Carpal tunnel |
| #2 | Median nerve |
| #3 | Median neuropathy |
| #4 | Median neuropathies |
| #5 | Median neuritis |
| #6 | Carpal tunnel syndrome/exp |
| #7 | #1 OR #2 OR #3 OR #4 OR #5 OR #6 |
| #8 | Entrapment |
| #9 | Compress |
| #10 | Compressive |
| #11 | Compression |
| #12 | Focal |
| #13 | #8 OR #9 OR #10 OR #11 OR #12 |
| #14 | Neuropathy |
| #15 | Neuropathies |
| #16 | Neuritis |
| #17 | #14 OR #15 OR #16 |
| #18 | #13 AND #17 |
| #19 | #7 OR #18 |
| #20 | Inject* |
| #21 | Steroid |
| #22 | Steroids |
| #23 | Corticosteroid |
| #24 | Corticosteroids |
| #25 | Betamethasone |
| #26 | Triamcinolone acetonide |
| #27 | Methylprednisolone |
| #28 | #21 OR #22 OR #23 OR #24 OR #25 OR #26 OR #27 |
| #29 | Dextrose |
| #30 | Glucose |
| #31 | D5W |
| #32 | Prolotherapy |
| #33 | Prolotherapy/exp |
| #34 | #29 OR #30 OR #31 OR #32 OR #33 |
| #35 | Platelet rich |
| #36 | PRP |
| #37 | Autologous |
| #38 | Platelet rich plasma/exp |
| #39 | #35 OR #36 OR #37 OR #38 |
| #40 | Ozone |
| #41 | Ozone/exp |
| #42 | #40 OR #41 |
| #43 | Hyaluronic |
| #44 | Hyaluronic acid/exp |
| #45 | #43 OR #44 |
| #46 | Hyalase |
| #47 | Progesterone |
| #48 | #20 OR #28 OR #34 OR #39 OR #42 OR #45 OR #46 OR #47 |
| #49 | #19 AND #48 |

| Database | Search terms for query |
| --- | --- |
| **Cochrane Library (started from 1971)** | Search for articles published from database inception to May 10, 2023. |
| #1 | Carpal tunnel |
| #2 | Median nerve |
| #3 | Median neuropathy |
| #4 | Median neuropathies |
| #5 | Median neuritis |
| #6 | [Carpal tunnel syndrome] explode all trees |
| #7 | #1 OR #2 OR #3 OR #4 OR #5 OR #6 |
| #8 | Entrapment |
| #9 | Compress |
| #10 | Compressive |
| #11 | Compression |
| #12 | Focal |
| #13 | #8 OR #9 OR #10 OR #11 OR #12 |
| #14 | Neuropathy |
| #15 | Neuropathies |
| #16 | Neuritis |
| #17 | #14 OR #15 OR #16 |
| #18 | #13 AND #17 |
| #19 | #7 OR #18 |
| #20 | Inject* |
| #21 | Steroid |
| #22 | Steroids |
| #23 | Corticosteroid |
| #24 | Corticosteroids |
| #25 | Betamethasone |
| #26 | Triamcinolone acetonide |
| #27 | Methylprednisolone |
| #28 | #21 OR #22 OR #23 OR #24 OR #25 OR #26 OR #27 |
| #29 | Dextrose |
| #30 | Glucose |
| #31 | D5W |
| #32 | Prolotherapy |
| #33 | [Prolotherapy] explode all trees |
| #34 | #29 OR #30 OR #31 OR #32 OR #33 |
| #35 | Platelet rich |
| #36 | PRP |
| #37 | Autologous |
| #38 | [Platelet rich plasma] explode all trees |
| #39 | #35 OR #36 OR #37 OR #38 |
| #40 | Ozone |
| #41 | [Ozone] explode all trees |
| #42 | #40 OR #41 |
| #43 | Hyaluronic |
| #44 | [Hyaluronic acid] explode all trees |
| #45 | #43 OR #44 |
| #46 | Hyalase |
| #47 | Progesterone |
| #48 | #20 OR #28 OR #34 OR #39 OR #42 OR #45 OR #46 OR #47 |
| #49 | #19 AND #48 |
