# Supplementary material for: Injection therapy for carpal tunnel syndrome: A systematic review and network meta-analysis of randomized controlled trials
Source: PLoS One. 2024 May 16;19(5):e0303537. doi: 10.1371/journal.pone.0303537 (PMC11098370; doi:10.1371/journal.pone.0303537)
Supplement: S1 File — (DOCX) [file pone.0303537.s002.docx]

| **Table 1. Characteristics of included trials.** | | | | | | | | | |
| --- | --- | --- | --- | --- | --- | --- | --- | --- | --- |
| Author, year | CTS severity | Group | Injectants | Patient number | Mean age (years) | Mean symptom duration (months) | Short-term follow-up duration | Long-term follow-up duration | Outcomes |
| Wu et al.^36^ | Mild to moderate | Dextrose | 5 mL of 5% dextrose | 30 | 58.47 | 44.57 | 1 month | 6 months | BCTQ-SSS score,  BCTQ-FSS score, pain score, SNCV, and DML |
|  |  | Placebo | 5 mL of normal saline | 30 | 58.10 | 44.47 |  |  |  |
| Wu et al.^34^ | Mild to moderate | Dextrose | 5 mL of 5% dextrose | 27 | 58.6 | 46.8 | 1 month | 6 months | BCTQ-SSS score,  BCTQ-FSS score, pain score, SNCV, and  DML |
|  |  | Steroid | 3 mL of triamcinolone acetonide plus 2 mL of normal saline | 27 | 54.3 | 45.6 |  |  |  |
| Elawamy et al.^30^ | Mild to moderate | Hyalase | Hyalase powder dissolved in 10 mL of saline solution | 30 | 40.7 | 8.5 | 1 month | 6 months | BCTQ-SSS score,  BCTQ-FSS score, pain score, SNCV, and  DML |
|  |  | Placebo | 10 mL of saline solution | 30 | 38.3 | 8.5 |  |  |  |
| Kamel et al.^33^ | Mild to moderate | Insulin | 10 IU of Neutral Protamine Hagedorn insulin | 20 | 40.7 | 8.5 | 10 weeks | N/A | BCTQ-SSS score,  BCTQ-FSS score, SNCV, and  DML |
|  |  | Steroid | 40 mg of methylprednisolone | 20 | 44.7 | 8.1 |  |  |  |
| Su et al.^25^ | Mild to moderate | Hyaluronic acid | 2.5 mL (25 mg) of hyaluronic acid | 17 | 50.9 | 35.6 | 1 month | 6 months | BCTQ-SSS score,  BCTQ-FSS score, pain score,  SNCV, and  DML |
|  |  | Placebo | 2.5 mL of normal saline | 15 | 58.9 | 28.6 |  |  |  |
| Forogh et al.^27^ | Mild to moderate | Ozone | 3 mL (30 μg) of ozone plus 1 mL of lidocaine | 20 | 54.70 | 9.10 | 6 weeks | 12 weeks | BCTQ-SSS score,  BCTQ-FSS score, pain score, CMAP, and  SNAP |
|  |  | Steroid | 40 mg of triamcinolone acetonide plus 1 mL of lidocaine | 20 | 53.65 | 10.82 |  |  |  |
| Ginanneschi et al.^39^ | Minimal to mild | 17-Alpha-hydroxyprogesterone | 0.5 mL (85 mg) of 17-alpha-hydroxyprogesterone | 8 | 47 | N/A | 1 month | 6 months | BCTQ-SSS score,  BCTQ-FSS score, pain score, DML, and  CMAP |
|  |  | Steroid | 0.5 mL (20 mg) of triamcinolone acetonide | 8 |  |  |  |  |  |
| Chen et al.^28^ | Bilateral moderate to severe | Platelet-rich plasma | 3.5 mL of platelet-rich plasma | 24 | 53 | 35.3 | 1 month | 12 months | BCTQ-SSS score,  BCTQ-FSS score, pain score, SNCV, and  DML |
|  |  | Placebo | 3.5 mL of normal saline | 24 |  | 36.2 |  |  |  |
| Shen et al.^31^ | Moderate | Dextrose | 3 mL of 5% dextrose | 26 | 58.5 | 37.5 | 1 month | 6 months | BCTQ-SSS score,  BCTQ-FSS score, SNCV, and DML |
|  |  | Platelet-rich plasma | 3 mL of platelet-rich plasma | 26 | 56.8 | 58.3 |  |  |  |
| Senna et al.^32^ | Mild to moderate | Platelet-rich plasma | 2 mL of platelet-rich plasma | 43 | 38.3 | N/A | 1 month | 3 months | BCTQ-SSS score,  BCTQ-FSS score, pain score, SNCV,  DML,  CMAP, and  SNAP |
|  |  | Steroid | 1 mL (40 mg) of methylprednisolone acetate | 42 | 40.7 | N/A |  |  |  |
| Hashim et al.^29^ | Mild to moderate | Platelet-rich plasma | 1 mL of platelet-rich plasma plus 0.5 mL of 1% lidocaine | 20 | 48.8 | 24.1 | 1.5 months | 3 months | BCTQ-SSS score,  BCTQ-FSS score, pain score, and DML |
|  |  | Steroid | 1 mL (40 mg) of methylprednisolone acetate plus 0.5 mL of 1% lidocaine | 20 | 49.15 | 24.34 |  |  |  |
| Armstrong et al.^42^ | N/A | Steroid | 1 mL (6 mg) of betamethasone | 43 | 51.9 | N/A | 2 weeks | N/A | BCTQ-SSS score,  BCTQ-FSS score, and DML |
|  |  | Placebo | 1 mL of normal saline | 38 | 51.2 | N/A |  |  |  |
| Peters-Veluthamaningal et al.^41^ | N/A | Steroid | 1 mL (10 mg) of triamcinolone acetonide | 36 | 56.5 | 13 weeks (median) | 1 month | 12 months | BCTQ-SSS score and BCTQ-FSS score |
|  |  | Placebo | 1 mL of normal saline | 33 | 57.6 | 26 weeks (median) |  |  |  |
| Karadas et al.^40^ | Except normal | Steroid | 40 mg of triamcinolone acetonide plus 4 mL of 1% procaine HCl | 33 | 46.35 | 9.4 | 2 months | 6 months | Pain score,  SNCV,  DML, and  CMAP |
|  |  | Placebo | 4 mL of 1% procaine HCl | 32 | 46.75 | 10.25 |  |  |  |
| Karadas et al.^38^ | Except normal | Steroid | 40 mg of triamcinolone acetonide | 20 | 46.40 | 9.46 | 2 months | 6 months | BCTQ-SSS score,  BCTQ-FSS score, pain score, SNCV,  DML,  CMAP, and  SNAP |
|  |  | Placebo | 1 mL of 0.09% saline | 19 | 48.40 | 9.86 |  |  |  |
| Atroshi et al.^37^ | Normal to moderate | Steroid | 1 mL (40 mg) of methylprednisolone plus 1 mL of saline plus 1 mL of lidocaine | 37 | 44 | N/A | 10 weeks | N/A | BCTQ-SSS score |
|  |  | Placebo | 2 mL of saline plus 1 mL of lidocaine | 37 | 49 | N/A |  |  |  |
| Roghani et al.^35^ | Moderate | Steroid | 1 mL (40 mg) of triamcinolone plus 1 mL of normal saline plus 1 mL of 2% lidocaine | 32 | 66 | N/A | 2 weeks | 6 months | DML and  SNAP |
|  |  | Placebo | 2 mL of normal saline plus 1 mL of 2% lidocaine | 30 | 63.4 | N/A |  |  |  |
| Hofer et al.^26^ | Normal to moderate | Steroid | 1 mL (40 mg) of triamcinolone plus 1 mL of normal saline plus 1 mL of lidocaine | 37 | 44 | N/A | N/A | 5 years | BCTQ-SSS score |
|  |  | Placebo | 2 mL of normal saline plus 1 mL of lidocaine | 37 | 49 | N/A |  |  |  |
| CTS, carpal tunnel syndrome; mL, milliliter; mg: milligram; IU, International Unit; μg, microgram; BCTQ-FSS, Boston Carpal Tunnel Syndrome Questionnaire Functional Status Scale; BCTQ-SSS, Boston Carpal Tunnel Syndrome Questionnaire Symptom Severity Scale; SNCV, sensory nerve conductive velocity; DML, distal motor latency; CMAP, compound muscle action potential; SNAP, sensory nerve action potential; N/A, not applicable. | | | | | | | | | |

| **Table 2. PEDro scores** | | | | | | | | | | | | | |
| --- | --- | --- | --- | --- | --- | --- | --- | --- | --- | --- | --- | --- | --- |
|  | 1* | 2 | 3 | 4 | 5 | 6 | 7 | 8 | 9 | 10 | 11 | Total | Rating |
| Armstrong et al.^42^ | V | V |  | V | V | V | V | V | V | V | V | 9 | Excellent |
| Atroshi et al.^37^ | V | V | V | V | V | V | V | V |  | V | V | 9 | Excellent |
| Chen et al.^28^ | V | V |  | V | V | V | V | V |  | V | V | 8 | Good |
| Elawamy et al.^30^ | V | V |  | V | V |  | V | V | V | V | V | 8 | Good |
| Forogh et al.^27^ | V | V | V | V | V | V | V |  | V | V | V | 9 | Excellent |
| Ginanneschi et al.^39^ |  | V |  | V |  |  |  | V |  | V | V | 5 | Fair |
| Hashim et al.^29^ | V | V |  | V |  |  | V | V | V | V | V | 7 | Good |
| Hofer et al.^26^ | V | V | V | V | V | V | V | V | V | V | V | 10 | Excellent |
| Kamel et al.^33^ |  | V |  | V | V |  |  |  | V | V | V | 6 | Good |
| Karadas et al.^40^ | V | V |  | V | V | V | V | V | V | V | V | 9 | Excellent |
| Karadas et al.^38^ | V | V |  | V | V | V | V | V | V | V | V | 9 | Excellent |
| Peters-Veluthamaningal et al.^41^ | V | V | V | V | V | V | V | V | V | V | V | 10 | Excellent |
| Roghani et al.^35^ |  | V | V | V | V | V | V | V | V | V | V | 10 | Excellent |
| Senna et al.^32^ | V | V | V | V |  |  | V | V | V | V | V | 8 | Good |
| Shen et al.^31^ | V | V |  | V |  |  | V | V | V | V | V | 7 | Good |
| Su et al.^25^ | V | V |  | V | V | V | V | V |  | V | V | 8 | Good |
| Wu et al.^36^ | V | V |  | V | V | V | V | V | V | V | V | 9 | Excellent |
| Wu et al.^34^ | V | V |  | V | V | V | V | V | V | V | V | 9 | Excellent |
| PEDro scale criteria: 1, eligibility criteria and source of participants; 2, random allocation; 3, concealed allocation; 4, baseline comparability; 5, blinded participants; 6, blinded therapists; 7, blind assessors; 8, adequate follow-up; 9, intention-to-treat analysis; 10, between-group comparisons; 11, point estimates and variability. *Not included in calculation of total score. | | | | | | | | | | | | | |

| **Table 3. Network meta-analysis results: short-term changes in BCTQ-SSS score** | | | | | | | | |
| --- | --- | --- | --- | --- | --- | --- | --- | --- |
| Pairwise meta-analysis | | | | | | | | |
| **Platelet-rich plasma** | - | - | - | −0.38 [−0.83; 0.08] | −0.25 [−0.92; 0.42] | - | - | −0.61 [−1.31; 0.09] |
| 0.04 [−0.78; 0.85] | **Insulin** | - | - | −0.28 [−1.01; 0.46] | - | - | - | - |
| −0.06 [−0.88; 0.75] | −0.10 [−1.14; 0.94] | **Ozone** | - | −0.18 [−0.91; 0.56] | - | - | - | - |
| −0.17 [−1.08; 0.74] | −0.21 [−1.34; 0.93] | −0.11 [−1.24; 1.03] | **Hyaluronic acid** | - | - | - | - | −0.75 [−1.57; 0.07] |
| −0.24 [−0.60; 0.12] | −0.28 [−1.01; 0.46] | −0.18 [−0.91; 0.56] | −0.07 [−0.94; 0.80] | **Steroid** | 0.34 [−0.33; 1.00] | - | −0.54 [−1.61; 0.54] | −0.83 [−1.16; −0.51] |
| −0.26 [−0.71; 0.19] | −0.30 [−1.14; 0.55] | −0.20 [−1.04; 0.64] | −0.09 [−1.01; 0.83] | −0.02 [−0.44; 0.39] | **Dextrose** | - | - | −0.31 [−0.95; 0.33] |
| −0.39 [−1.14; 0.37] | −0.42 [−1.44; 0.60] | −0.32 [−1.34; 0.70] | −0.21 [−1.26; 0.83] | −0.15 [−0.85; 0.56] | −0.12 [−0.90; 0.65] | **Hyalase** | - | −0.53 [−1.18; 0.11] |
| −0.78 [−1.90; 0.35] | −0.81 [−2.11; 0.49] | −0.71 [−2.01; 0.59] | −0.60 [−1.98; 0.77] | −0.54 [−1.61; 0.54] | −0.51 [−1.66; 0.64] | −0.39 [−1.67; 0.89] | **17-Alpha-hydroxyprogesterone** | - |
| −0.92 [−1.31; −0.53] | −0.95 [−1.74; −0.17] | −0.86 [−1.64; −0.07] | −0.75 [−1.57; 0.07] | −0.68 [−0.96; −0.39] | −0.66 [−1.08; −0.23] | −0.53 [−1.18; 0.11] | −0.14 [−1.25; 0.96] | **Placebo** |
| Network meta-analysis | | | | | | | | |
| Data are expressed as SMDs [95% CIs]. Significant results are underlined.  SMD, standard mean difference; CI, credible interval; BCTQ-SSS, Boston Carpal Tunnel Syndrome Questionnaire Symptom Severity Scale. | | | | | | | | |

| **Table 4. Assessment of inconsistency among studies: short-term changes in BCTQ-SSS score** | | | | | | | | |
| --- | --- | --- | --- | --- | --- | --- | --- | --- |
| Comparison | Number of studies | Network meta-analysis | Direct | Indirect | Difference | Lower limit of 95% CI | Upper limit of 95% CI | *P* value |
| Dextrose vs. placebo | 1 | −0.6562 | −0.3073 | −0.9293 | 0.621 | −0.2339 | 1.4778 | 0.1543 |
| Dextrose vs. platelet-rich plasma | 1 | 0.2626 | 0.2481 | 0.2743 | −0.0262 | −0.9271 | 0.8747 | 0.9546 |
| Dextrose vs. steroid | 1 | 0.0227 | −0.3371 | 0.2573 | −0.5944 | −1.4471 | 0.2586 | 0.1720 |
| Platelet-rich plasma vs. placebo | 1 | −0.9188 | −0.6124 | −1.0619 | 0.4496 | −0.3956 | 1.2948 | 0.2972 |
| Steroid vs. placebo | 4 | −0.6789 | −0.833 | −0.1258 | −0.7072 | −1.3988 | −0.0156 | 0.0451 |
| Platelet-rich plasma vs. steroid | 2 | −0.23 | −0.3774 | −0.0161 | −0.3612 | −1.1002 | 0.3777 | 0.338 |
| CI, credible interval; BCTQ-SSS, Boston Carpal Tunnel Syndrome Questionnaire Symptom Severity Scale. | | | | | | | | |

| **Table 5. Network meta-analysis results: long-term changes in BCTQ-SSS score** | | | | | | | |
| --- | --- | --- | --- | --- | --- | --- | --- |
| Pairwise meta-analysis | | | | | | | |
| **Hyalase** | - | - | - | - | - | −2.29 [−2.94; −1.64] | - |
| −1.55 [−2.31; −0.79] | **Dextrose** | 0.25 [−0.30; 0.79] | - | - | −1.04 [−1.61; −0.47] | −0.69 [−1.21; −0.17] | - |
| −1.56 [−2.32; −0.81] | −0.02 [−0.39; 0.35] | **Platelet-rich plasma** | - | - | −0.60 [−0.96; −0.24] | −0.61 [−1.19; −0.03] | - |
| −1.76 [−2.72; −0.80] | −0.22 [−1.02; 0.59] | −0.20 [−1.00; 0.61] | **Hyaluronic acid** | - | - | −0.53 [−1.24; 0.18] | - |
| −1.79 [−3.04; −0.54] | −0.25 [−1.31; 0.82] | −0.23 [−1.27; 0.81] | −0.03 [−1.31; 1.25] | **17-Alpha-hydroxyprogesterone** | −0.44 [−1.44; 0.55] | - | - |
| −2.24 [−3.00; −1.48] | −0.69 [−1.07; −0.31] | −0.67 [−0.98; −0.37] | −0.47 [−1.28; 0.33] | −0.44 [−1.44; 0.55] | **Steroid** | −0.27 [−0.90; 0.36] | −0.23 [−0.85; 0.40] |
| −2.29 [−2.94; −1.64] | −0.74 [−1.13; −0.36] | −0.73 [−1.11; −0.34] | −0.53 [−1.24; 0.18] | −0.50 [−1.57; 0.57] | −0.05 [−0.45; 0.34] | **Placebo** | - |
| −2.46 [−3.44; −1.48] | −0.91 [−1.64; −0.19] | −0.90 [−1.59; −0.21] | −0.70 [−1.72; 0.32] | −0.67 [−1.84; 0.50] | −0.23 [−0.85; 0.40] | −0.17 [−0.91; 0.57] | **Ozone** |
| Network meta-analysis | | | | | | | |
| Data are expressed as SMDs [95% CIs]. Significant results are underlined.  SMD, standard mean difference; CI, credible interval; BCTQ-SSS, Boston Carpal Tunnel Syndrome Questionnaire Symptom Severity Scale. | | | | | | | |

| **Table 6. Assessment of inconsistency among studies: short-term changes in BCTQ-SSS score** | | | | | | | | |
| --- | --- | --- | --- | --- | --- | --- | --- | --- |
| Comparison | Number of studies | Network meta-analysis | Direct | Indirect | Difference | Lower limit of 95% CI | Upper limit of 95% CI | *P* value |
| Dextrose vs. placebo | 1 | −0.7446 | −0.6921 | −0.8099 | 0.1178 | −0.6621 | 0.8977 | 0.7672 |
| Dextrose vs. platelet-rich plasma | 1 | −0.0165 | 0.2481 | −0.2401 | 0.4881 | −0.2530 | 1.2293 | 0.1967 |
| Dextrose vs. steroid | 1 | −0.6898 | −1.0393 | −0.4183 | −0.6211 | −1.3786 | 0.1365 | 0.1081 |
| Platelet-rich-plasma vs. placebo | 1 | −0.7281 | −0.6124 | −0.8191 | 0.2067 | −0.567 | 0.9804 | 0.6005 |
| Steroid vs. placebo | 1 | −0.0549 | −0.2693 | 0.0809 | −0.3502 | −1.1562 | 0.4559 | 0.3945 |
| Platelet-rich-plasma vs. steroid | 2 | −0.6732 | −0.6034 | −0.8558 | 0.2524 | −0.4292 | 0.934 | 0.468 |
| CI, credible interval; BCTQ-SSS, Boston Carpal Tunnel Syndrome Questionnaire Symptom Severity Scale. | | | | | | | | |

| **Table 7. Network meta-analysis results: short-term changes in BCTQ-FSS score** | | | | | | | | |
| --- | --- | --- | --- | --- | --- | --- | --- | --- |
| Pairwise meta-analysis | | | | | | | | |
| **Hyalase** | - | - | - | - | - | - | - | −1.31 [−1.93; −0.68] |
| −0.25 [−1.23; 0.72] | **Insulin** | - | - | - | - | - | −0.58 [−1.27; 0.12] | - |
| −0.32 [−1.29; 0.65] | −0.07 [−1.04; 0.91] | **Ozone** | - | - | - | - | −0.51 [−1.20; 0.18] | - |
| −0.42 [−1.66; 0.81] | −0.17 [−1.41; 1.07] | −0.10 [−1.34; 1.13] | **17-Alpha-hydroxyprogesterone** | - | - | - | −0.41 [−1.43; 0.62] | - |
| −0.56 [−1.55; 0.43] | −0.31 [−1.38; 0.77] | −0.24 [−1.31; 0.83] | −0.14 [−1.45; 1.18] | **Hyaluronic acid** | - | - | - | −0.75 [−1.52; 0.02] |
| −0.62 [−1.36; 0.12] | −0.37 [−1.16; 0.42] | −0.30 [−1.09; 0.49] | −0.20 [−1.30; 0.90] | −0.06 [−0.93; 0.81] | **Dextrose** | 0.20 [−0.42; 0.81] | −0.31 [−0.91; 0.30] | −0.86 [−1.46; −0.26] |
| −0.70 [−1.43; 0.02] | −0.45 [−1.22; 0.31] | −0.38 [−1.15; 0.38] | −0.28 [−1.36; 0.80] | −0.14 [−1.00; 0.71] | −0.08 [−0.49; 0.33] | **Platelet-rich plasma** | −0.17 [−0.58; 0.24] | −0.20 [−0.84; 0.43] |
| −0.83 [−1.52; −0.14] | −0.58 [−1.27; 0.12] | −0.51 [−1.20; 0.18] | −0.41 [−1.43; 0.62] | −0.27 [−1.09; 0.55] | −0.21 [−0.59; 0.18] | −0.13 [−0.45; 0.20] | **Steroid** | −0.54 [−0.88; −0.20] |
| −1.31 [−1.93; −0.68] | −1.05 [−1.80; −0.31] | −0.99 [−1.74; −0.24] | −0.88 [−1.95; 0.19] | −0.75 [−1.52; 0.02] | −0.69 [−1.08; −0.29] | −0.60 [−0.97; −0.24] | −0.48 [−0.77; −0.19] | **Placebo** |
| Network meta-analysis | | | | | | | | |
| Data are expressed as SMDs [95% CIs]. Significant results are underlined.  SMD, standard mean difference; CI, credible interval; BCTQ-FSS, Boston Carpal Tunnel Syndrome Questionnaire Functional Status Scale. | | | | | | | | |

| **Table 8. Assessment of inconsistency among studies: short-term changes in BCTQ-FSS score** | | | | | | | | |
| --- | --- | --- | --- | --- | --- | --- | --- | --- |
| Comparison | Number of studies | Network meta-analysis | Direct | Indirect | Difference | Lower limit of 95% CI | Upper limit of 95% CI | *P* value |
| Dextrose vs. placebo | 1 | −0.6867 | −0.8575 | −0.5520 | −0.3055 | −1.1054 | 0.4945 | 0.4542 |
| Dextrose vs. platelet-rich plasma | 1 | −0.0829 | 0.1961 | −0.3080 | 0.5041 | −0.3192 | 1.3274 | 0.2301 |
| Dextrose vs. steroid | 1 | −0.2084 | −0.306 | −0.1414 | −0.1646 | −0.9507 | 0.6215 | 0.6815 |
| Platelet-rich plasma vs. placebo | 1 | −0.6038 | −0.2041 | −0.8065 | 0.6023 | −0.1741 | 1.3789 | 0.1284 |
| Steroid vs. placebo | 3 | −0.4782 | −0.5377 | −0.3157 | −0.2221 | −0.8745 | 0.4303 | 0.5047 |
| Platelet-rich plasma vs. steroid | 2 | −0.1256 | −0.1683 | −0.0507 | −0.1176 | −0.7962 | 0.561 | 0.7341 |
| CI, credible interval; BCTQ-FSS, Boston Carpal Tunnel Syndrome Questionnaire Functional Status Scale. | | | | | | | | |

| **Table 9. Network meta-analysis results: long-term changes in BCTQ-FSS score** | | | | | | | |
| --- | --- | --- | --- | --- | --- | --- | --- |
| Pairwise meta-analysis | | | | | | | |
| **Hyalase** | - | - | - | - | - | - | −3.83 [−4.72; −2.94] |
| −2.92 [−3.91; −1.93] | **Dextrose** | 0.37 [−0.23; 0.98] | - | - | - | −1.04 [−1.66; −0.41] | −1.11 [−1.71; −0.51] |
| −2.99 [−3.98; −2.01] | −0.07 [−0.48; 0.34] | **Platelet-rich plasma** | - | - | - | −0.61 [−1.01; −0.20] | −0.61 [−1.25; 0.02] |
| −3.10 [−4.54; −1.67] | −0.18 [−1.30; 0.94] | −0.11 [−1.20; 0.98] | **17-Alpha-hydroxyprogesterone** | - | - | −0.61 [−1.64; 0.43] | - |
| −3.22 [−4.42; −2.02] | −0.30 [−1.10; 0.50] | −0.23 [−0.99; 0.53] | −0.12 [−1.36; 1.12] | **Ozone** | - | −0.49 [−1.17; 0.19] | - |
| −3.47 [−4.63; −2.31] | −0.55 [−1.41; 0.31] | −0.48 [−1.34; 0.38] | −0.37 [−1.72; 0.98] | −0.25 [−1.35; 0.85] | **Hyaluronic acid** | - | −0.35 [−1.10; 0.39] |
| −3.71 [−4.70; −2.72] | −0.79 [−1.20; −0.37] | −0.72 [−1.06; −0.37] | −0.61 [−1.64; 0.43] | −0.49 [−1.17; 0.19] | −0.24 [−1.10; 0.62] | **Steroid** | −0.10 [−0.78; 0.57] |
| −3.83 [−4.72; −2.94] | −0.90 [−1.34; −0.47] | −0.83 [−1.26; −0.41] | −0.72 [−1.84; 0.40] | −0.60 [−1.41; 0.20] | −0.35 [−1.10; 0.39] | −0.12 [−0.55; 0.32] | **Placebo** |
| Network meta-analysis | | | | | | | |
| Data are expressed as SMDs [95% CIs]. Significant results are underlined.  SMD, standard mean difference; CI, credible interval; BCTQ-FSS, Boston Carpal Tunnel Syndrome Questionnaire Functional Status Scale. | | | | | | | |

| **Table 10. Assessment of inconsistency among studies: long-term changes in BCTQ-FSS score** | | | | | | | | |
| --- | --- | --- | --- | --- | --- | --- | --- | --- |
| Comparison | Number of studies | Network meta-analysis | Direct | Indirect | Difference | Lower limit of 95% CI | Upper limit of 95% CI | *P* value |
| Dextrose vs. placebo | 1 | −0.9024 | −1.1076 | −0.6735 | −0.4341 | −1.3093 | 0.4411 | 0.3310 |
| Dextrose vs. platelet-rich plasma | 1 | −0.0715 | 0.3721 | −0.4514 | 0.8235 | −0.0023 | 1.6493 | 0.0506 |
| Dextrose vs. steroid | 1 | −0.7869 | −1.0366 | −0.5852 | −0.4513 | −1.2904 | 0.3878 | 0.2918 |
| Platelet-rich-plasma vs. placebo | 1 | −0.8309 | −0.6124 | −1.0093 | 0.397 | −0.4573 | 1.2513 | 0.3624 |
| Steroid vs. placebo | 1 | −0.1155 | −0.105 | −0.1227 | 0.0177 | −0.8638 | 0.8992 | 0.9686 |
| Platelet-rich plasma vs. steroid | 2 | −0.7154 | −0.6056 | −0.9831 | 0.3774 | −0.3767 | 1.1315 | 0.3266 |
| CI, credible interval; BCTQ-FSS, Boston Carpal Tunnel Syndrome Questionnaire Functional Status Scale. | | | | | | | | |

| **Table 11. Network meta-analysis results: short-term changes in pain score** | | | | | | | |
| --- | --- | --- | --- | --- | --- | --- | --- |
| Pairwise meta-analysis | | | | | | | |
| **Platelet-rich-plasma** | −0.49 [−1.21; 0.23] | - | - | - | - | - | - |
| −0.49 [−1.21; 0.23] | **Steroid** | −0.09 [−1.39; 1.22] | 0.04 [−0.96; 1.05] | - | −0.34 [−1.40; 0.71] | - | −1.02 [−1.76; −0.28] |
| −0.57 [−2.06; 0.91] | −0.09 [−1.39; 1.22] | **17-Alpha-hydroxyprogesterone** | - | - | - | - | - |
| −0.69 [−1.75; 0.37] | −0.20 [−0.98; 0.58] | −0.11 [−1.63; 1.41] | **Dextrose** | - | - | - | −0.46 [−1.45; 0.54] |
| −0.78 [−2.25; 0.70] | −0.29 [−1.58; 1.00] | −0.20 [−2.03; 1.63] | −0.09 [−1.45; 1.27] | **Hyaluronic acid** | - | - | −0.60 [−1.71; 0.51] |
| −0.83 [−2.11; 0.44] | −0.34 [−1.40; 0.71] | −0.26 [−1.94; 1.42] | −0.15 [−1.46; 1.17] | −0.06 [−1.73; 1.61] | **Ozone** | - | - |
| −0.94 [−2.33; 0.45] | −0.45 [−1.64; 0.74] | −0.37 [−2.13; 1.40] | −0.25 [−1.52; 1.01] | −0.16 [−1.66; 1.33] | −0.11 [−1.70; 1.49] | **Hyalase** | −0.44 [−1.44; 0.56] |
| −1.38 [−2.35; −0.41] | −0.89 [−1.54; −0.24] | −0.81 [−2.26; 0.65] | −0.69 [−1.47; 0.09] | −0.60 [−1.71; 0.51] | −0.55 [−1.79; 0.70] | −0.44 [−1.44; 0.56] | **Placebo** |
| Network meta-analysis | | | | | | | |
| Data are expressed as SMDs [95% CIs]. Significant results are in underlined.  SMD, standard mean difference; CI, credible interval. | | | | | | | |

| **Table 12. Assessment of inconsistency among studies: changes in short-term pain score** | | | | | | | | |
| --- | --- | --- | --- | --- | --- | --- | --- | --- |
| Comparison | Number of studies | Network meta-analysis | Direct | Indirect | Difference | Lower limit of 95% CI | Upper limit of 95% CI | *P* value |
| Dextrose vs. placebo | 1 | −0.6924 | −0.4554 | −1.0637 | 0.6083 | −0.9896 | 2.2062 | 0.4556 |
| Dextrose vs. steroid | 1 | 0.198 | −0.0442 | 0.5642 | −0.6083 | −2.2062 | 0.9896 | 0.4556 |
| Steroid vs. placebo | 2 | −0.8903 | −1.0196 | −0.4113 | −0.6083 | −2.2062 | 0.9896 | 0.4556 |
| CI, credible interval. | | | | | | | | |

| **Table 13. Network meta-analysis results: long-term changes in pain score** | | | | | | | |
| --- | --- | --- | --- | --- | --- | --- | --- |
| Pairwise meta-analysis | | | | | | | |
| **Dextrose** | - | - | - | −1.15 [−2.31; 0.02] | - | - | −0.84 [−1.99; 0.30] |
| −0.02 [−1.23; 1.19] | **Platelet-rich plasma** | - | - | −0.60 [−1.42; 0.21] | - | - | - |
| −0.03 [−1.71; 1.65] | −0.01 [−1.65; 1.63] | **17-Alpha-hydroxyprogesterone** | - | −0.59 [−2.02; 0.83] | - | - | - |
| −0.22 [−1.68; 1.23] | −0.20 [−1.79; 1.38] | −0.19 [−2.17; 1.78] | **Hyalase** | - | - | - | −1.13 [−2.27; 0.02] |
| −0.62 [−1.52; 0.27] | −0.60 [−1.42; 0.21] | −0.59 [−2.02; 0.83] | −0.40 [−1.76; 0.97] | **Steroid** | - | −0.48 [−1.68; 0.71] | −0.99 [−1.82; −0.16] |
| −0.98 [−2.50; 0.54] | −0.96 [−2.61; 0.69] | −0.95 [−2.97; 1.07] | −0.75 [−2.44; 0.93] | −0.35 [−1.79; 1.08] | **Hyaluronic acid** | - | −0.37 [−1.60; 0.86] |
| −1.11 [−2.60; 0.38] | −1.09 [−2.53; 0.35] | −1.08 [−2.93; 0.78] | −0.88 [−2.70; 0.93] | −0.48 [−1.68; 0.71] | −0.13 [−1.99; 1.73] | **Ozone** | - |
| −1.35 [−2.24; −0.46] | −1.33 [−2.43; −0.23] | −1.32 [−2.92; 0.28] | −1.13 [−2.27; 0.02] | −0.73 [−1.46; 0.01] | −0.37 [−1.60; 0.86] | −0.24 [−1.64; 1.16] | **Placebo** |
| Network meta-analysis | | | | | | | |
| Data are expressed as SMDs [95% CIs]. Significant results are in underlined.  SMD, standard mean difference; CI, credible interval. | | | | | | | |

| **Table 14. Assessment of inconsistency among studies: long-term changes in pain scores** | | | | | | | | |
| --- | --- | --- | --- | --- | --- | --- | --- | --- |
| Comparison | Number of studies | Network meta-analysis | Direct | Indirect | Difference | Lower limit of 95% CI | Upper limit of 95% CI | *P* value |
| Dextrose vs. placebo | 1 | −1.3488 | −0.8445 | −2.139 | 1.2945 | −0.5334 | 3.1223 | 0.1651 |
| Dextrose vs. steroid | 1 | −0.6233 | −1.1479 | 0.1466 | −1.2945 | −3.1223 | 0.5334 | 0.1651 |
| Steroid vs. placebo | 2 | −0.7254 | −0.9911 | 0.3034 | −1.2945 | −3.1223 | 0.5334 | 0.1651 |
| CI, credible interval. | | | | | | | | |
