# Supplementary material for: Injection therapy for carpal tunnel syndrome: A systematic review and network meta-analysis of randomized controlled trials
Source: PLoS One. 2024 May 16;19(5):e0303537. doi: 10.1371/journal.pone.0303537 (PMC11098370; doi:10.1371/journal.pone.0303537)
Supplement: S2 File — (DOCX) [file pone.0303537.s003.docx]

**Figure Legends of S1 Figures**

**Fig 1. Flowchart of article selection.**

**Fig 2. Short-term changes in SNCV.** (A) Network diagram displaying at least one placebo-controlled trial for each injectant, except for insulin. (B) Forest plot indicating that steroids yielded significantly more favorable outcomes than placebo.

**Fig 3. Short-term changes in SNAP.** (A) Network diagram indicating that only steroids were compared with placebo. (B) Forest plot indicating no significant difference among the injectants.

**Fig 4. Short-term changes in DML.** (A) Network diagram revealing at least one placebo-controlled trial for each injectant, except for insulin and 17-alpha-hydroxyprogesterone. (B) Forest plot indicating no significant difference among the injectants.

**Fig 5. Short-term changes in CMAP.** (A) Network diagram indicating that only steroids were compared with placebo. (B) Forest plot indicating no significant difference among the injectants.

**Fig 6. Long-term changes in SNCV.** (A) Network diagram indicating that all the injectants were compared with placebo. (B) Forest plot indicating that dextrose, hyalase, platelet-rich plasma and steroids yielded significantly more favorable outcomes than placebo.

**Fig 7. Long-term changes in SNAP.** (A) Network diagram indicating that only steroids were compared with placebo. (B) Forest plot indicating no significant difference among the injectants.

**Fig 8. Long-term changes in DML.** (A) Network diagram displaying at least one placebo-controlled trial for each injectant, except for 17-alpha-hydroxyprogesterone. (B) Forest plot indicating that hyalase yielded significantly more favorable outcomes than placebo.

**Fig 9. Long-term changes in CMAP.** (A) Network diagram indicating that only steroids were compared with placebo. (B) Forest plot indicating no significant difference among the injectants.


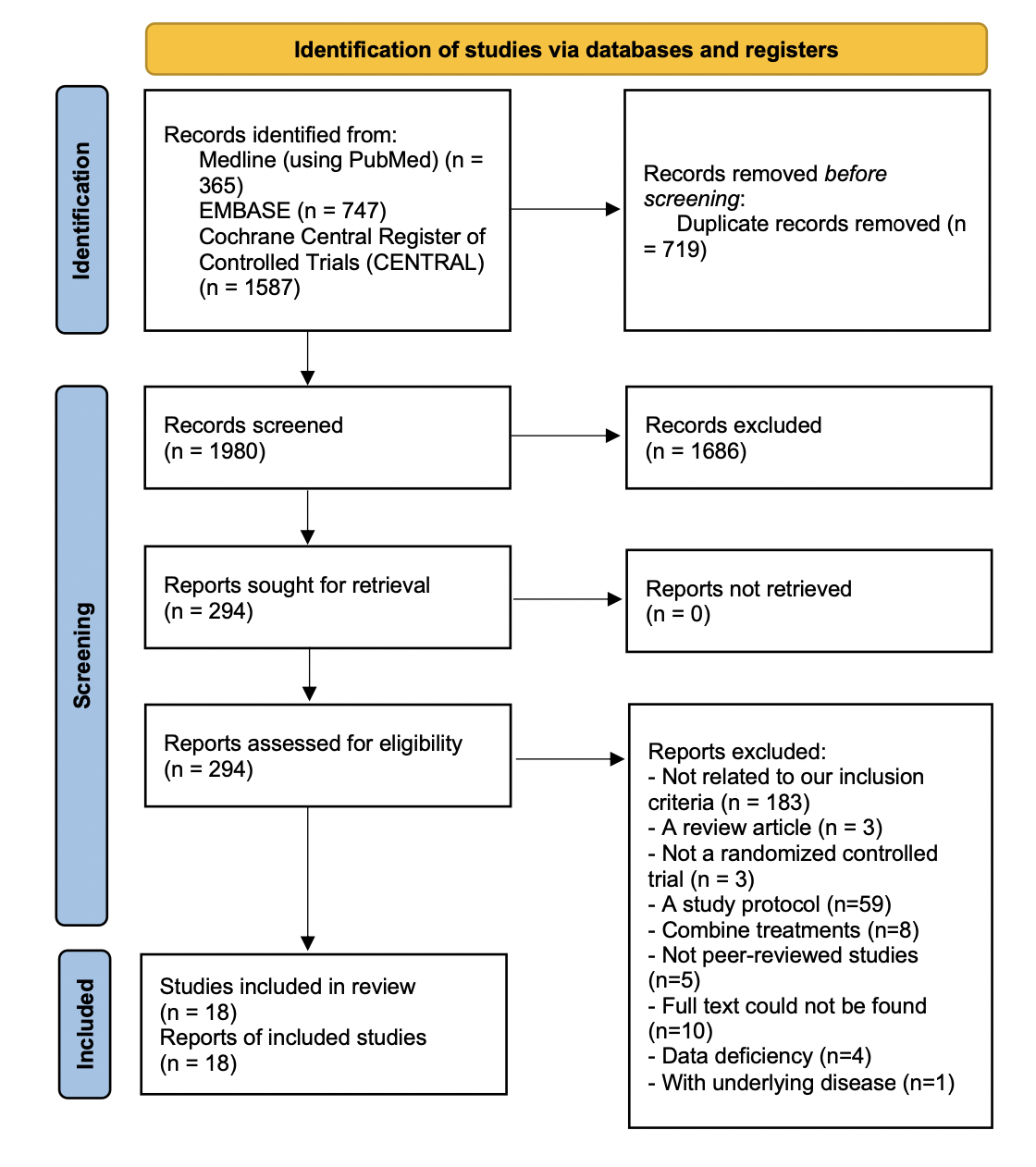


**Fig 1. Flowchart of article selection.**

**Fig 2. Short-term changes in SNCV.** (A) Network diagram displaying at least one placebo-controlled trial for each injectant, except for insulin. (B) Forest plot indicating that steroids yielded significantly more favorable outcomes than placebo. SMD, standard mean difference; CI, credible interval; SNCV, sensory nerve conductive velocity.

**Fig 3. Short-term changes in SNAP.** (A) Network diagram indicating that only steroids were compared with placebo. (B) Forest plot indicating no significant difference among the injectants. SMD, standard mean difference; CI, credible interval; SNAP, sensory nerve action potential.

**Fig 4. Short-term changes in DML.** (A) Network diagram revealing at least one placebo-controlled trial for each injectant, except for insulin and 17-alpha-hydroxyprogesterone. (B) Forest plot indicating no significant difference among the injectants. SMD, standard mean difference; CI, credible interval; DML, distal motor latency.

**Fig 5. Short-term changes in CMAP.** (A) Network diagram indicating that only steroids were compared with placebo. (B) Forest plot indicating no significant difference among the injectants. SMD, standard mean difference; CI, credible interval; CMAP, compound muscle action potential.

**Fig 6. Long-term changes in SNCV.** (A) Network diagram indicating that all the injectants were compared with placebo. (B) Forest plot indicating that dextrose, hyalase, platelet-rich plasma and steroids yielded significantly more favorable outcomes than placebo. SMD, standard mean difference; CI, credible interval; SNCV, sensory nerve conductive velocity.

**Fig 7. Long-term changes in SNAP.** (A) Network diagram indicating that only steroids were compared with placebo. (B) Forest plot indicating no significant difference among the injectants. SMD, standard mean difference; CI, credible interval; SNAP, sensory nerve action potential.

**Fig 8. Long-term changes in DML.** (A) Network diagram displaying at least one placebo-controlled trial for each injectant, except for 17-alpha-hydroxyprogesterone. (B) Forest plot indicating that hyalase yielded significantly more favorable outcomes than placebo. SMD, standard mean difference; CI, credible interval; DML, distal motor latency.

**Fig 9. Long-term changes in CMAP.** (A) Network diagram indicating that only steroids were compared with placebo. (B) Forest plot indicating no significant difference among the injectants. SMD, standard mean difference; CI, credible interval; CMAP, compound muscle action potential.
